# Research Buddy partnership in a MD–PhD program: lessons learned

**DOI:** 10.1186/s40900-023-00414-9

**Published:** 2023-02-18

**Authors:** Daniel J. Gould, Marion Glanville-Hearst, Samantha Bunzli, Peter F. M. Choong, Michelle M. Dowsey

**Affiliations:** 1 Department of Surgery, St. Vincent’s Hospital, University of Melbourne, Melbourne, VIC Australia; 2 School of Health Sciences and Social Work, Griffith University, Nathan Campus, Brisbane, QLD Australia; 3 Physiotherapy Department, Royal Brisbane and Women’s Hospital, Brisbane, QLD Australia; 4 Department of Orthopaedics, St. Vincent’s Hospital, Melbourne, VIC Australia

**Keywords:** Patient, Partnership, Involvement, Co-design, Research Buddy

## Abstract

**Background and aims:**

There is increasing recognition of the importance of patient involvement in research. In recent years, there has also been growing interest in patient partnerships with doctoral studies students. However, it can be difficult to know where to start and how to go about such involvement activities. The purpose of this perspective piece was to share experiential insight of the experience of a patient involvement program such that others can learn from this experience.

**Body:**

This is a co-authored perspective piece centred on the experience of MGH, a patient who has had hip replacement surgery, and DG, a medical student completing a PhD, participating in a Research Buddy partnership over the course of over 3 years. The context in which this partnership took place was also described to facilitate comparison with readers’ own circumstances and contexts. DG and MGH met regularly to discuss, and work together on, various aspects of DG’s PhD research project. Reflexive thematic analysis was conducted on reflections from DG and MGH regarding their experience in the Research Buddy program to synthesise nine lessons which were then corroborated with reference to published literature on patient involvement in research. These lessons were: learn from experience; tailor the program; get involved early; embrace uniqueness; meet regularly; build rapport; ensure mutual benefit; broad involvement; regularly reflect and review.

**Conclusions:**

In this perspective piece, a patient and a medical student completing a PhD reflected upon their experience co-designing a Research Buddy partnership within a patient involvement program. A series of nine lessons was identified and presented to inform readers seeking to develop or enhance their own patient involvement programs. Researcher-patient rapport is foundational to all other aspects of the patient’s involvement.

## Plain English summary

The importance of patient involvement in research is gaining recognition. Existing research centres, as well as those that are just getting started, need to find their own way to involve patients and community members. However, learning from the experience of others is crucial to ensure every effort is made to do this in a fruitful way. Therefore, we aimed to share our experience and provide a list of lessons learned to help other researchers and patients get started and work together effectively. Our research centre developed a framework for involving patients in joint replacement research. Part of this framework is a ‘Research Buddy’ program, where a research student partners with a patient so that the research they conduct is more relevant and applicable to the target population. In our case, the research student partnered with someone who had a hip replacement to develop and test a questionnaire for an interview study about artificial intelligence in shared decision-making. The student and patient worked together and wrote this perspective piece outlining nine lessons so readers can learn from their experience of this program. The lessons were: learn from experience, tailor the program, get involved early, embrace uniqueness, meet regularly, build rapport, ensure mutual benefit, broad involvement, regularly reflect and review. People interested in starting, or improving, their own patient involvement activities can learn from our experience. These lessons will need to be adapted to fit the purpose and unique situation of other researchers and patients who have different needs and circumstances.

## Introduction

### Background

There is growing recognition of the importance of patient and public involvement and engagement in research [1, 2]. However, it can be difficult to know where to begin and how to go about it. Sharing experiential knowledge is critical for different groups seeking to develop and deploy their own programs and strategies [3]. This is because a one-size-fits-all approach will not address the culture and context of the unique settings in which these programs take place [4–7]. Rather, strategies should be tailored to each unique setting [8]. An in-depth description of the experience of the patient involvement program in a particular context, including the nature of the program and the challenges overcome, provides others with ideas and examples they can modify to suit their own needs [1, 3, 9]. These other groups can read about the experience of others and possibly avoid some pitfalls while harnessing the strengths of the program. This is not intended to restrict creativity nor to provide a prescriptive roadmap, but rather to provide an experiential account of the nature of engagement in a particular setting and offer suggestions for ways in which engagement can take place.

In recent years, there has also been growing interest in efforts to involve patients in doctoral studies [5, 10–17]. This presents a unique opportunity to instil recognition of the importance of patient involvement early in the career of researchers and equip them with the skills and experience to facilitate meaningful involvement. It simultaneously offers patients an opportunity to partner with researchers in this formative stage of their career as they develop their research interests and approach to research.

### Aims and rationale

The aim of this perspective piece was for the first two members (DG and MGH) of a Research Buddy partnership (described below in the ‘Context’ section) to critically reflect on their experience of this involvement activity throughout the course of a MD–PhD program.

Through this reflective process, lessons learned through experience were consolidated such that they can be of benefit to others seeking to learn from this experiential insight as they establish or improve their own patient involvement programs [1, 3, 18–20]. We also provided a detailed description of the context in which this Research Buddy partnership took place, to provide readers with a more complete understanding of the way in which this context influenced the partnership such that readers can consider the nature of their own unique situation and how this might impact upon patient involvement efforts [21].

### Overview of structure of this perspective piece

Definitions.

Theoretical Underpinnings.

Context.

Methods.

- Method of Patient Involvement throughout MD–PhD program
- Method of reflection in this perspective piece

Findings—Lessons Learned.

### Discussion

Conclusions

### Definitions

The body of literature in the patient involvement space is fraught with complex and nuanced terminology [22]. Therefore, the purpose of this section is to clarify the terminology and definitions used. Influential stakeholder involvement literature, as well as recent publications, were reviewed to select widely-used and functionally relevant terminology for this perspective piece [1, 22–33]. The other critical aspect of the rationale for selecting these terms and definitions was that they were agreed upon with the patient partner, MGH, as they accurately described the nature of her involvement.

Terms used in this paper include the following: patient, involvement, partnership, and co-design.

The term ‘patient’ was used because this perspective piece concerns involvement with patients as distinct from members of the public. While this term may invoke the image of an individual seeking care, in the context of involvement it also refers to individuals with experience of a health condition and its management [28], which in this case is total joint replacement surgery.

Building upon this is the definition of ‘involvement’, which is commonly accepted to refer to research done ‘with’ or ‘by’ patients rather than ‘for’, ‘to’, or ‘about’ them [30]. One specific type of involvement is the ‘patient partnership’, in which patients are embedded in various research activities at different levels and in multiple stages of the research process [1]. A particularly powerful example of a patient partnership is the Research Buddy program, which has been described previously as a program in which a doctoral student works closely with a patient throughout their project [13] and which was also described and reflected upon later in this perspective piece in the section ‘Method of Patient Involvement throughout MD–PhD program’.

Finally, the term ‘co-design’ was used in this piece to describe the active, voluntary process of researcher and patient partner working together on the design of a PhD project and its constituent studies, as this terminology is appropriate for the timeframe in which doctoral studies typically take place [22, 32–35].

### Theoretical underpinnings

Theory development was not an aim of this perspective piece. However, it is important to describe the theoretical underpinnings of the patient involvement program.

There are three main approaches to patient involvement in research: epistemological, consequentialist, and emancipatory or moral [33, 36, 37]. The epistemological approach recognises the fact that patients have lived experience of a health condition that can be of benefit to the research by broadening the perspective of researchers. The emancipatory approach recognises that patients have a right to be involved in publicly funded research that impacts upon them or the care they receive, and consequently researchers have a responsibility to involve patients. Finally, the consequentialist argument states that involvement can improve the design, conduct, and reporting of research.

Primarily, the emancipatory argument motivated the formation of the involvement program described in this perspective piece. Furthermore, patient involvement can lead to more ethically sound research [38], and this was recognised in the work reflected upon in this piece as MGH assisted DG in the ethics application process for the PhD research projects through document review. However, it was also recognised that the quality and relevance of research conducted at the research centre could be improved through involvement of a patient with lived experience of joint replacement surgery. Therefore, the consequentialist and epistemological arguments also underpinned the involvement program.

Recent literature has also advocated for a pragmatic approach to patient-oriented research by utilising patient involvement to ultimately improve health outcomes for affected individuals [39]. These authors recognised that achieving such impact is a complex task and, as such, mixed methods approaches to research are encouraged to harness the strength of both quantitative and qualitative research methodologies. This is particularly relevant to the current perspective piece, which describes patient involvement in a mixed methods PhD project on a topic of clinical relevance aimed to improve outcomes for patients.

Finally, the word ‘perspective’ was intentionally selected instead of the term ‘representative’ because it is unrealistic and unfair to ask a single patient with their own unique experience to generalise their experience to represent that of all patients with the same health condition. Rather, patient involvement was incorporated to broaden the perspective of the researchers while they co-designed research with the patient [36, 40].

### Context

A detailed description of the context in which the patient involvement program took place facilitates comparison to one’s own context such that the potential applicability of a framework and experience can be considered in detail [6, 7]. This is not intended to limit the generalisability of the findings reflected upon in this perspective piece. Rather, the specific context in which the patient involvement partnership and activities took place is described in detail such that readers can accurately compare this context to their own.

The patient involvement program described in this perspective piece took place in an Australian National Health and Medical Research Council Centre for Research Excellence for Total Joint Replacement, seeking to *O*ptimise *P*atient o*U*tcomes and *S*election for Total Joint Replacement (OPUS). OPUS is embedded in a hospital in Melbourne, Victoria, which is a tertiary referral centre for total joint replacement (TJR), providing care for people from a broad range of geographical regions, socioeconomic backgrounds, and cultures [41]. OPUS brings together clinician-researchers with backgrounds in orthopaedic surgery, nursing, and physiotherapy, as well as trialists, epidemiologists, health economists, statisticians and qualitative researchers. It comprises researchers at various stages of their careers, including graduate research students.

**Fig. 1:**
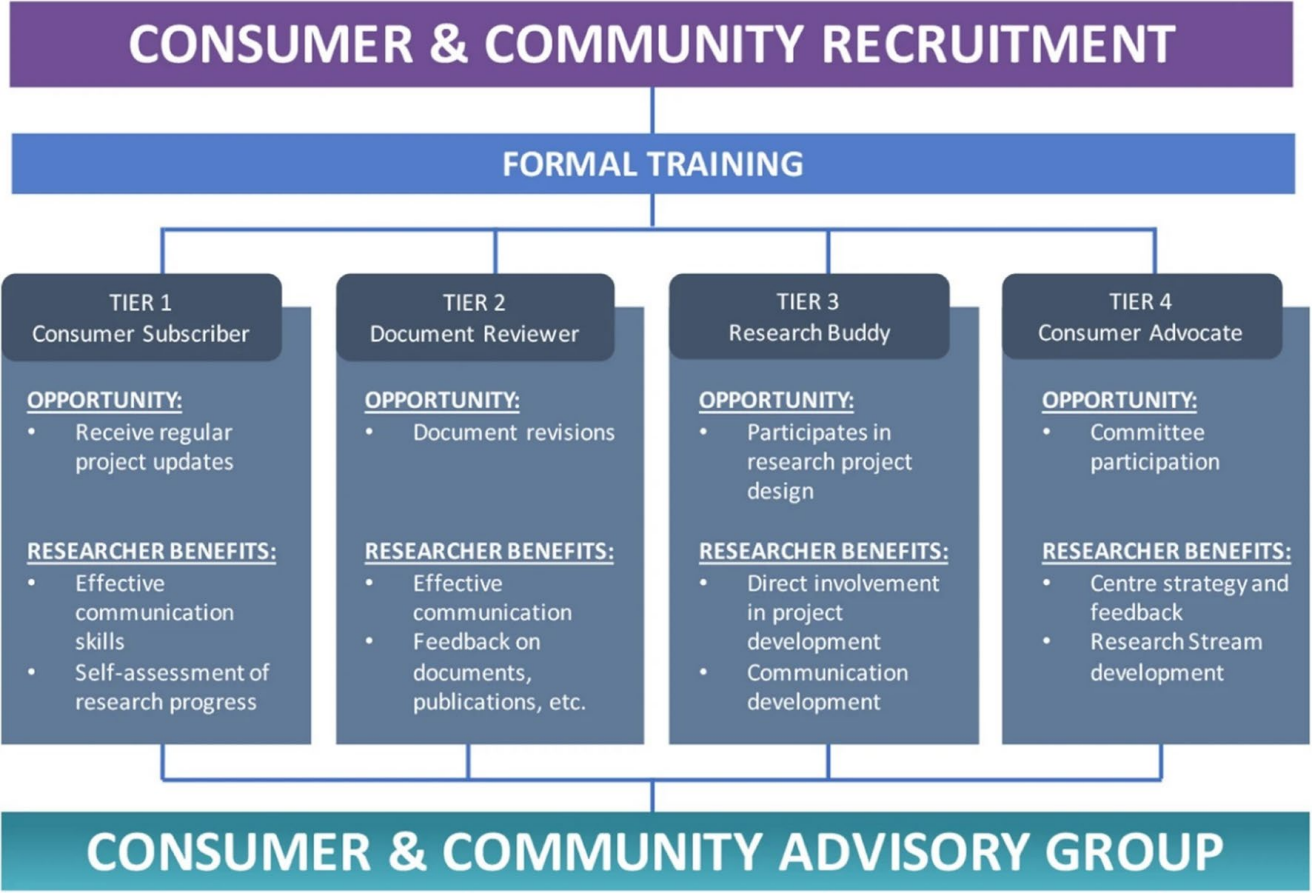
Proposed level of involvement for consumers and community members presents different opportunities of participation and the relevant benefits to researchers

The patient involvement program at OPUS was developed and launched in 2020 and has been described previously [42]. The program involves a four-tiered framework of involvement (see Fig. 1). Table 3 of this prior publication also outlines the remuneration fee schedule according to which MGH was remunerated for her time. This prior publication used the term ‘consumer’, therefore, for consistency, this terminology is used below when describing the framework.

This Figure was made available under the Creative Commons Attribution 4.0 (http://creativecommons.org/licenses/by/4.0/).

The Consumer and Community Advisory Group (bottom of Fig. 1) oversees the operations of the CCI program. It is chaired by a consumer (MGH, co-author on this paper).

This perspective piece details the experience of a Research Buddy, Tier 3 as shown in Fig. 1, and an MD–PhD candidate at OPUS. An MD–PhD candidate is a medical student who takes time out from their clinical studies to complete a PhD. The details of this Research Buddy program are described in the next section.

## Methods

### Method of patient involvement throughout MD–PhD program

The Research Buddy program reflected upon in this piece took the approach of broadening the perspective of the research team by including the voice a patient with lived experience of a health condition [43]. This is distinct from an approach which would seek to achieve representativeness from one patient partner on behalf of all patients with a shared lived experience, which would be unrealistic [31, 44].

The focus of this piece is on the Research Buddy partnership comprising authors DG and MGH. In this section, DG and MGH reflected upon their expectations prior to the commencement of the Research Buddy program and how their actual experience played out.

DG was a final-year postgraduate medical student in 2019, carrying out a research project on risk factors for readmission following total knee replacement (TKR) surgery. The opportunity arose to enrol in a PhD to expand upon this work, which led to the deferment of DG’s final year of medical school in order to complete a PhD as part of an MD–PhD program [45]. Building upon the research into risk factors for readmission following TKR, the PhD centred around the development of a clinical risk prediction tool utilising machine learning. DG, along with his PhD supervisors (authors MD, SB, and PC) felt that the strong clinical focus of the MD–PhD program would position it well for close involvement with a Research Buddy. MGH had previously participated, as a subject, in a study conducted by MD and PC investigating the impact of a mindfulness program on recovery post-surgery for patients undergoing total hip replacement (THR) [46]. MGH reached out to MD regarding the findings of the mindfulness study, coincidentally while MD was establishing the patient involvement framework at OPUS and seeking volunteers for the Research Buddy role. MGH subsequently embraced the opportunity and volunteered to be DG’s Research Buddy approximately 6 months after the official commencement of the PhD. MGH decided to take part specifically in DG’s project because she wanted to learn more about research, had a keen interest in artificial intelligence and machine learning and its application to lower limb joint replacement surgery, and has had a lifelong interest and involvement in academia. This presented a tangible opportunity to make a substantial impact on the direction of the research and cement its clinical, stakeholder-focused approach, and MGH felt her interests and experience aligned most closely with DG’s project [47].

During this formative stage of the PhD project, MGH and DG worked together to define the scope and structure of the Research Buddy program. There was a shared expectation of open-ended discussions and regular contact with updates on the research. Neither DG nor MGH had been involved in such a patient involvement partnership before, therefore they did not have rigid expectations about the manner in which the partnership would unfold. It was decided that regular monthly meetings would facilitate the development of rapport, ongoing discussion of ideas for the PhD project and opportunities for involvement its constituent studies, and the opportunity for regular updates on progress.

The first part of DG’s PhD project discussed in these meetings was the systematic review, which was in the manuscript drafting stage and benefited greatly from MGH’s input on the clarity of the language [48, 49] and ensuring the pertinence and importance of the topic were clearly communicated. Patient involvement in systematic reviews has been reported extensively in the literature [32, 50–52], and it was an opportunity for MGH to gain familiarity with the topic at the foundation of the PhD project. More broadly, the regular monthly meetings were critical in defining the overall scope and purpose of the PhD project. Specifically, during the early stages it was difficult to determine how to make the research more clinically relevant. MGH had a background in occupational therapy and medical anthropology and had previously been involved in qualitative interview projects in which she interviewed participants and coded transcripts. The idea of a qualitative study exploring the views of TKR patients on the use of artificial intelligence and machine learning for risk prediction in shared clinical decision-making was already being developed, and MGH felt this was a fitting opportunity to be involved given her personal journey as a patient and her prior academic and research experience. MGH was involved as a co-author on the qualitative study and made a substantial contribution by pilot-testing the interview guide, contributing to the reflexive thematic analysis, and preparing and editing the manuscript for publication.

Next, MGH reviewed all of the ethics approval application documents for the studies comprising DG’s PhD project. Not only did this improve the clarity of the documents in a similar way to MGH’s review of the systematic review manuscript, but it also led to the development of more ethically conscious research by incorporating a patient’s perspective into the application [38] and helped DG to clarify the way these studies related to one another and formed a cohesive program of research.

Beyond the specific elements in which MGH made a direct contribution, DG’s reflection upon the stakeholder involvement in the form of the Research Buddy program prompted a shift in focus from machine learning to enhance predictive performance of the TKR readmission prediction model, to stakeholder involvement and engagement in the whole process of developing clinical risk prediction models, including not only patients but also clinicians [29, 53]. In light of this, thanks to MGH’s Research Buddy input, DG designed the PhD project with stakeholder involvement embedded throughout.

At the time of writing this perspective piece, the main studies of the PhD project had been completed and DG was in the final stages of thesis write-up following feedback provided by MGH on the first full draft. The overall focus of the PhD project is on co-design with stakeholders, inspired by the Research Buddy partnership with MGH and cemented by exploration of the literature [24, 54–56].

The PhD project centres around the development of a machine learning model to predict 30-day readmission [57] in people undergoing TKA surgery years [58, 59] for osteoarthritis (OA) [60], co-designed with clinicians and patients [61]. The project is divided into four components, each comprising at least one main study. While MGH’s involvement in the project overall has been outlined above, what follows is a brief description of each part of the PhD project as well as the level [29] and phase [62] at which MGH was involved. While a high level of involvement was aimed at from the early phases of each part of the PhD project, it is recognised that higher levels of involvement are not automatically more desirable [27] and, since some aspects of the project were already underway when MGH and DG commenced the Research Buddy program, flexibility and responsiveness to the realities of the circumstances were of utmost importance in deciding with MGH at what level she wanted to be involved.

1. Identify and appraise risk factors for 30-day readmission following TKA.
  - As previously mentioned, MGH was involved in reviewing the final draft of the systematic review manuscript [63] and provided feedback on the clarity of the language. The next stage of this part of the project involved a modified Delphi and focus group study involving clinicians who care for TKR patients to gain their insight on the clinical relevance of risk factors identified in the systematic review and meta-analysis, as well as giving them the opportunity to suggest novel risk factors which had not been identified in prior literature. MGH helped by observing the pilot test of the focus group and providing feedback on its structure and conduct. This study has also been published [64].
    i. Level = Involve
    ii. Phase = Design and preparation, dissemination
2. Develop and internally validate the model.
  - The findings of the systematic review and meta-analysis, as well as the Delphi and focus group study, were utilised alongside machine learning to develop a clinically applicable risk prediction tool for 30-day readmission following TKR which harnessed both clinical reasoning and statistical learning techniques. The manuscript is currently under review in an academic journal. MGH and DG discussed the project in their regular meetings as DG designed the study, particularly the purpose of the predictive tool and the way in which it could be used in the live clinical setting to facilitate shared clinical decision-making between patient and surgeon. and MGH reviewed several drafts of the manuscript ahead of submission for publication.
    i. Level = Involve
    ii. Phase = Dissemination
3. Explore the understanding of AI for risk prediction in shared clinical decision-making among people with TKA.
  - This is the qualitative semi-structured interview study, involving interviews with 20 total knee replacement patients, in which MGH had the most direct involvement, being included as a co-author. Patient and stakeholder involvement in qualitative studies is a longstanding practice [5, 7, 65–69]. MGH was involved in refining the research question and interview guideline through two rounds of pilot-testing. MGH was also actively involved in the reflexive thematic analysis and in drafting the manuscript for submission to an academic journal, where it is currently under review.
    i. Level = Collaborate
    ii. Phase = Design and preparation, conduct and implementation, and dissemination
4. Compare the performance of the predictive model to that of clinicians involved in the care of people receiving TKA surgery.
  - Preliminary findings for this study have been analysed and recruitment is almost complete at the time of writing this perspective piece. MGH was involved in a similar way to the predictive model development study, however this comparison study involved recruiting clinicians to participate in a survey. MGH reviewed recruitment material and discussed with DG how to structure the online survey to ensure the instructions were clear and straightforward and the length of the survey was manageable for busy clinicians.
    i. Level = Involve
    ii. Phase = Design and preparation, dissemination

### Method of reflection in this perspective piece

Rather than speaking for the patient, it is important for the patient to speak for themselves. Prior reflective work on patient involvement in doctoral research adopted this approach to great effect [5, 14]. This piece was therefore co-written by MGH and DG, the two members of the inaugural Research Buddy partnership at OPUS.

Pertinent elements of the GRIPP2 (Guidance for Reporting Involvement of Patients and the Public) checklist [70] were adhered to while writing this piece.

The experiential insight gained by DG and MGH through their involvement in the Research Buddy program was consolidated in regular meetings throughout DG’s PhD project. Additionally, DG and MGH conducted an informal interview using questions informed by prior literature in which patient advocates from various patient involvement programs in the UK were interviewed [9]. This interview was audio recorded and transcribed. The transcript was then analysed using reflective thematic analysis [71] in which themes were generated inductively by DG and MGH based on MGH’s responses to the questions in the interview, informed by their shared experience in the Research Buddy partnership. These themes were then synthesised and refined into lessons, inspired by prior patient involvement literature which has used thematic analysis in a similar way to generate lessons and recommendations [1, 3, 55, 62, 72, 73].

A literature search was then carried out to identify literature reviews which contained lessons from, or recommendations for, patient involvement [1, 5, 13, 15, 28, 35, 62, 74, 75]. These reviews were used to corroborate the findings from our own inductive analysis and reflection.

DG and MGH derived these lessons by reflecting primarily on their experience in the Research Buddy program. However, MGH was embedded in various other patient involvement activities at the research centre (OPUS), therefore some of these lessons will be broadly applicable beyond Research Buddy partnerships. This will depend on the patient’s desired level of involvement, and it is advised that readers are flexible in their interpretation and implementation of these findings.

### Findings: lessons learned

#### Lesson (1) learn from experience

Don’t go in completely blind. Learn from the experience of others as much as possible. Bring in experienced patient partners, and researchers experienced in patient involvement, who can offer advice on promising avenues to explore as well as flagging those which are less likely to be fruitful. For example, as part of the Research Buddy program reflected upon in this perspective piece, Carol Vleeskens was consulted to conduct a training session attended by both MGH and DG. Carol was a co-author on the publication outline OPUS’ patient involvement framework [42] and has extensive experience in patient involvement [76]. MGH was then involved in preparing an orientation day for prospective patient partners at OPUS, which she also attended.

Another powerful resource is published work on the experience and appraisal of other researcher-patient partnerships sharing their experience [5].

This lesson is pertinent to consider both in preparation for partnering with patients in order to maximise the likelihood of success prior to launching involvement programs [8, 13, 48, 77], as well as for ongoing appraisal and improvement of existing programs based on patient partner feedback [1].

#### Lesson (2) tailor the program

A one-size-fits-all approach does not work. Patient involvement programs should be bespoke. They should be tailored to the unique context of the research centre engaging the patients [5, 78]. Learning from the work of others is critical for generating ideas about how one’s own program could take shape [78], but it is equally important to modify these programs so they are fit for purpose.

Tailoring the patient involvement program has been emphasised in prior literature as a critical component of fruitful involvement through the translation of general principles into context-specific systems and actions [4, 5, 8, 15, 35, 74].

#### Lesson (3) get involved early

If possible, involve patients in discussions where ideas are generated for research projects. Allow ample time to include patients on ethics applications and governance approvals to ensure they can make a meaningful contribution to the research.

Integrating patient involvement in earlier stages of research helps to facilitate truly patient-led research. Based on the experience outlined in this perspective piece, there are two main ways in which this can be done. First, patient involvement can be embedded in the ongoing work of established research centres as well as the commencement of work at new research centres in order to identify research priorities and steer the direction of research activities [4, 7]. Second, in the style of Research Buddy partnerships, patients can be partnered with PhD students in order to co-design the program of research in a meaningful way by embedding engagement and co-design at the beginning of the student’s project [19]. It is not difficult to imagine a pipeline in which researchers collaborate with patients to generate ideas and then these ideas are translated into PhD research projects with patients.

Having patients engaged at these different stages of idea-generation and research project design could have the added benefit of anticipating and addressing challenges likely to arise in complex research projects such that solutions can be suggested proactively [29].

Early involvement has been demonstrated or recommended numerous times in a diverse range of contexts and for different levels of involvement [5, 7, 15, 28, 35, 43, 75].

#### Lesson (4) embrace uniqueness

Patients bring a wealth of unique experience. They should be treated as individuals with their own unique characteristics, experience, and insight [79]. Embrace their unique skills and experience. Throughout regular meetings and correspondence, various aspects of the patient’s experience may enable them to bring a unique perspective to the research which researchers could not have anticipated.

Embracing uniqueness of patient partners has been found to encourage more inclusive stakeholder involvement by demonstrating a flexibility in the researchers and responsiveness to the individual needs, experiences, and preferences of people seeking to be involved [1, 5, 35, 48, 77].

#### Lesson (5) meet regularly

Meet regularly, right from the start, even in the absence of a clear idea of what to do in terms of exactly how the patient will be involved. The level and nature of involvement can be collaboratively defined through these regular meetings.

Be open to a variety of forms the patient’s involvement could take and be willing to engage in some trial and error. Some ideas will not pan out, and that is just part of the process. Be willing to persevere and adapt. For example, during the qualitative study conducted as part of DG’s PhD project, MGH attempted cross-coding of the qualitative interview transcripts using the coding framework being developed by DG and author SB. However, MGH found that her style of coding based her prior experience was not functioning as well as it had for previous projects. DG and MGH attempted different styles and continued to discuss their findings in their regular monthly meetings, however ultimately this cross-coding exercise was abandoned as it was becoming quite convoluted and MGH felt more comfortable discussing the transcripts and contributing to the discussions around the development of themes through reflexive thematic analysis.

Both DG and MGH had an openness that resulted in these meetings being fruitful beyond the pragmatic aspects of setting the research agenda and tracking progress; they were critical in building rapport. Even when there were no specific research updates, the regular meeting time was maintained and utilised for an opportunity to discuss topics related to the research.

Regular meetings are a critical component of impactful patient involvement programs reported in the literature [15, 35, 62, 75] as they facilitates ongoing communication, continuous feedback, and strong rapport-building as expanded upon in Lesson 6.

#### Lesson (6) build rapport

Establishing trust and rapport is critical to the success of the program. Put in the time. Create an environment in which frank and open discussions are encouraged. Follow through on action items from meetings and email discussions. Respect the patient’s autonomy while supporting them in their role. Listen to the patient and take their feedback into account. Demonstrate to the patient how their input is influencing the research. This designated time to meet and talk openly was critical for building rapport, which is arguably the most important component of patient involvement to increase the likelihood of success [37, 80]. The centrality of rapport to the overall success of patient involvement in research is reflected in the large volume of diverse literature recognising its importance [1, 5, 17, 28, 30, 35, 62, 73, 75, 81–84].

#### Lesson (7) ensure mutual benefit

Patients are more engaged when there is something in it for them. Ensure the interaction is mutually beneficial. For example, patients might learn more about their condition, or the condition of those they care for, through the patient involvement program [85].

This pragmatic aspect of involvement has been recognised in the literature detailing meaningful and impactful involvement activities that recognise the importance of ensuring the involvement benefits the research, the researchers, and the patient partners themselves [1, 19, 35, 62, 75, 83].

The nature of a successful patient involvement program, in which there is rapport, mutual respect, and open discussion, is associated with a certain degree of passive learning simply by the two parties being in contact with one another [85]. Without any specific educational component to the program, regular and open communication regarding a common focus in the context of a research centre or program can have the effect of educating the patient about research [53], and the researcher about the patient’s lived experience of their condition [72, 86]. Patients may also learn more about their own condition, its management, and current research being done to further understanding about it [85].

#### Lesson (8) broad involvement

It is beneficial to have the patient embedded in the broader patient involvement activities at the research centre, not just the Research Buddy (or equivalent) program. This may enable the patient to participate in broad discussions about current research and setting the direction of future research.

This is important for contextualising the Research Buddy’s work and enhancing their understanding of the way in which it fits into the broader body of work being conducted at the research centre in which the program is taking place. This is not to say patient partners should be stretched beyond their limit, but rather that they are not forced to limit themselves to one project and instead are encouraged to broaden their perspective through opportunities to be involved in varying capacities in other research projects and activities depending on their interests and capacity [28, 35, 82].

#### Lesson (9) regularly reflect and review

It is not enough to have good intentions. Having a framework is also critical, but the patient’s role may evolve over time and ethical considerations related to their involvement also evolve. It is easy for the patient’s role to devolve into tokenism despite the best intentions [22]. Regular meetings incorporating feedback from patient partners on their involvement in the research are critical to ensuring the patient’s role as partner in the research is respected and maintained. This requires transparency on the part of the researcher regarding how the patient’s involvement is influencing the research. Researchers must also be open to feedback from patients that may indicate they are not satisfied with their involvement and adjustments to their involvement may be required.

One potential way in which the patient partnership can break down is for researchers to treat them less like partners in the research process and more like participants in quasi-qualitative research by simply calling upon patients to complete surveys or answer questions as if in a qualitative interview. This is problematic because patient involvement in research is distinct from qualitative research and must be treated as such, but vigilance and effort is required to maintain the boundaries between the two [22].

Beyond this specific problem, regular reflection and review are broadly applicable to every aspect of patient involvement to ensure the patient’s involvement is meeting expectations as these expectations evolve throughout the research project alongside changes in the patient’s capacity and interests.

Regular review is crucial to ensure ethical principles are being adhered to at all times as the patient’s role evolves. Circumstances change, both intentionally and unintentionally, so it is critical to have discussions specifically dedicated to reviewing the patient’s role and ensuring they are still meaningfully involved and ethical principles are adhered to [87]. There may be occasions on which the patient’s preconceived ideas need to be discussed and worked through.

Building upon the other lessons outlined in this perspective piece, rapport based on mutual respect, coupled with regular meetings, provide the structure and nurture the culture necessary to facilitate continuous feedback as well as regular designated touch points at which reflection and review can take place [5, 13, 28, 35, 62, 74, 75, 83, 87].

## Discussion

### Summary of findings

In this perspective piece, a patient partner with lived experience of total joint replacement surgery (MGH) and a MD–PhD candidate (DG) reflected on their experience in a Research Buddy program throughout a PhD project. Their aim was to provide experiential insight such that other researchers and patient partners can learn from their experience as they develop and refine their own patient involvement programs. Inductive thematic analysis was applied to the transcript of an informal interview carried out between DG and MGH, informed by reflections from regular meetings throughout their Research Buddy partnership. Through this process, nine lessons were synthesised from their experience: Learn from experience, Tailor the program, Get involved early, Embrace uniqueness, Meet regularly, Build rapport, Ensure mutual benefit, Broad involvement, and Regularly reflect and review. These lessons were corroborated by published literature, and rapport was found to be the most important factor in successful patient involvement. Each of the remaining lessons either stem from and/or contribute to the development of rapport.

### Contribution to the literature

To the best of the authors’ knowledge, this is the first perspective piece on patient partnership in a MD–PhD program. This builds upon prior work highlighting the positive impact of partnering medical students with patients to further their clinical education and broaden their perspective, while empowering patients through the opportunity to learn more about their own health condition and health service experience while sharing their experiential insight with future clinicians in a formative stage of their career [88, 89]. It builds upon this by demonstrating how MD–PhD students, being situated at the interface between the research and clinical domains, can partner with patients in a fruitful way that has a meaningful impact upon their clinical development while also influencing the design, conduct, and reporting of the research. This was particularly noteworthy during DG’s PhD journey, most of which was undertaken during lockdowns imposed in light of the COVID-19 pandemic. Regular contact with MGH was, for long periods of time, DG’s only patient interaction and therefore was crucial to maintaining patient contact [88] from a clinical perspective as well as working with MGH to develop solutions to convert various research activities to be entirely online [15, 16].

Through regular meetings based on mutual trust and respect, largely comprising open discussion especially when there were no major research progress updates, building rapport was prioritised from the outset of the Research Buddy program. Since MGH and DG had the freedom to define the scope and nature of the program, they were able to emphasise the importance of this reciprocal relationship both through conversation as well as through document review, providing comments alongside DG’s PhD supervisors, which enabled MGH to provide a critical perspective on DG’s work. With strong rapport as the foundation and critical feedback encouraged throughout the research journey, MGH’s role as ‘critical friend’ was characterised and strengthened [10, 17].

This perspective piece also builds upon exemplary prior work reporting a Research Buddy program [13] by being co-authored with the Research Buddy. This perspective piece conveyed lessons learned through a program which drew on elements of embedded consultation [5, 13] and demonstrated flexibility in the level of involvement in various aspects of the PhD project in response to changes in MGH’s interests and capacity throughout the partnership [16].

Furthermore, while the PhD project reflected upon in this perspective piece did not strictly pertain to a digital health innovation nor to a full translational research innovation, a predictive model was developed which was intended for use in the clinical setting for shared clinical decision-making in orthopaedics. Therefore, MGH’s involvement throughout the project was critical for in laying a solid foundation for the development of a clinically relevant digital health innovation [90, 91] primed for translation into the clinical setting [27] in orthopaedics, which is a clinical discipline in which patient involvement is gaining recognition and appreciation [26].

### Implications of findings

The most impactful implication of the findings of this paper is that the description of the context in which the researcher-patient partnership took place, coupled with the co-authored lessons learned and experiential insight [3] offered through reflection by both members of the Research Buddy partnership, can be utilised by others [92, 93]. Learning through experience and reporting these lessons along with a detailed description of the context in which they were learned [6, 18] is an impactful outcome of its own [92]. This enables readers to inform their own involvement efforts, both when establishing involvement programs and refining them.

Through involvement, patients like MGH learn more about their health condition as well as the research that goes into the developments in management approaches and the way in which patients experience the healthcare system [85].

It is hoped that these findings can be of assistance to ongoing [11, 12] and future patient involvement efforts to bring researchers and patients onto the same page from the outset of research projects [49].

### Future directions

#### Evaluating impact

The question of how best to evaluate impact of patient involvement is longstanding and complicated [48, 77]. There have been many attempts over the years to answer this question [11, 12, 23, 73, 78, 79, 94–99], but it appears there is no single best approach to measuring the effectiveness, success, or impact of involvement [100], and perhaps such a measure will never exist. However, ongoing efforts to develop and validate consistent patient involvement evaluation measures are encouraged to facilitate the comparison of different involvement strategies and gain some indication of which might be the most promising in a given context, when resources are limited and ought to be allocated to the most promising strategy [101].

#### Exploring patient involvement in decision-making non-research patient involvement and empowerment

The research project reflected upon in this perspective piece centred around the development of a risk prediction tool intended to be used in the process of shared clinical decision-making. This perspective piece explored patient involvement in clinically-oriented research being carried out by a MD–PhD candidate, which positions it well for ongoing work exploring the role patients play in the shared clinical decision-making process [102–104], particularly as it pertains to the use of decision aids including predictive tools.

### Further reading

Further reading is recommended for those seeking to expand their knowledge of different types of patient involvement in varied contexts in order to better understand how to initiate, or improve, their own patient involvement programs [32, 40, 105–148].

### Strengths and limitations

Strengths of this perspective piece include the consistent and frequent contact between DG and MGH throughout the course of the PhD program, which facilitated widespread and deep involvement in various aspects of the research project and its constituent studies. This also facilitated continuous feedback and reflection through open discussion, which laid the foundation for the writing of this piece.

One potential limitation is that MGH may be regarded as a ‘professional’ patient partner, given her academic background. This may raise concerns about whether her perspective as a patient is diminished by her familiarity with academia and the research process. This was certainly an important consideration, especially considering MGH’s involvement spanned over 3 years and involved close and regular contact with DG. However, it was not a concern because regardless of how familiar MGH becomes with the research process, she will never lose her perspective as a patient who has lived experience of total joint replacement surgery [149].

## Conclusions

In this perspective piece, a patient and a medical student completing a PhD reflected upon their experience co-designing a Research Buddy partnership within a patient involvement program. To the authors’ knowledge, this is the first time such a partnership has been written about within the context of a MD–PhD program, which is uniquely placed at the interface between the research and clinical domains. A series of nine lessons was identified and presented to inform readers seeking to develop or enhance their own patient involvement programs: learn from experience, tailor the program, get involved early, embrace uniqueness, meet regularly, build rapport, ensure mutual benefit, broad involvement, regularly reflect and review. Researcher-patient rapport is foundational to all other aspects of the patient’s involvement.

## Data Availability

Not applicable.

## Abbreviations

PhD: Doctor of philosophy
MD: Doctor of medicine
GRIPP2: Guidance for reporting Involvement of Patients and the Public
OPUS: OPtimise Patient oUtcomes and Selection for Total Joint Replacement
TKR: Total knee replacement
THR: Total hip replacement
